# Surgical treatment of Killian‐Jamieson diverticulum: A case report and literature review

**DOI:** 10.1002/ccr3.2249

**Published:** 2019-06-04

**Authors:** Vagner Birk Jeismann, Edno Tales Bianchi, Sérgio Szachnowicz, Francisco Carlos Bernal da Costa Seguro, Francisco Tustumi, Andre Fonseca Duarte, Rubens Antonio Aissar Sallum, Ivan Cecconello

**Affiliations:** ^1^ Hospital das Clínicas, Faculty of Medicine University of São Paulo Cerqueira Cesar Sao Paulo Brazil

**Keywords:** deglutition disorders, esophageal diseases, esophageal diverticulum, esophageal motility disorders

## Abstract

This study describes a patient with symptomatic Killian‐Jamieson, a rare entity, successfully treated by cervical approach with diverticulum resection and esophagomyotomy.

## 1 INTRODUCTION

The Killian‐Jamieson diverticulum (KJD) is a rare disorder characterized by an outpouching from the lateral wall of the proximal cervical esophagus. The etiopathogenesis and treatment management are still unclear. This case report describes a successful surgical treatment with the cervical transcutaneous approach with diverticulum resection and associated esophagomyotomy of a patient with symptomatic KJD.

The KJD is defined as an outpouching from the lateral wall of the proximal cervical esophagus. These diverticula are, in fact, false diverticula and protrude through a muscular gap inferior to the cricopharyngeus muscle and superior‐lateral to the longitudinal muscle of the esophagus.^1^ This gap was first described by Killian and later by Jamieson as corresponding to the area where the recurrent laryngeal nerve enters the pharynx.^2^, ^3^ KJD is a rare disease as compared with Zenker's diverticulum (ZD), which is the most common diverticulum of this region. A ZD develops at the anatomically weak posterior region just above the cricopharyngeal muscle (the Killian's triangle).^4^, ^5^, ^6^

The pathogenesis of KJD is unclear, and the diagnosis is established by radiographic evaluation.^1^ The literature about KJD and its management is still scarce. Surgery is usually recommended for symptomatic KJD, although successful endoscopic treatment has been published.^4^, ^7^, ^8^

This case report describes a successful surgical treatment of a patient with symptomatic KJD. The available literature about this disease was reviewed.

## 2 CASE REPORT

**Figure 1:**
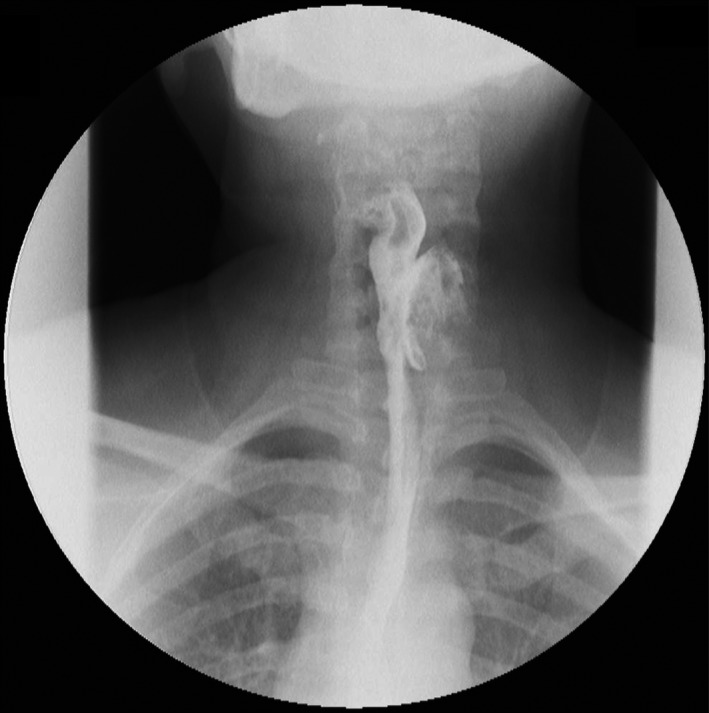
The barium swallow study showing a 4 cm Killian‐Jamieson diverticulum arising anteriorly and left‐laterally from the cervical esophagus

**Figure 2:**
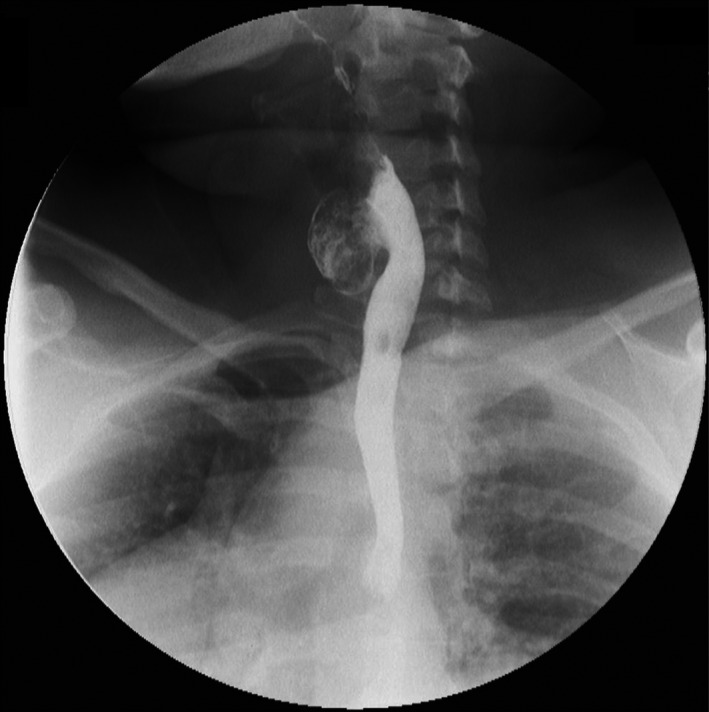
The barium swallow study showing Killian‐Jamieson diverticulum

A 48‐year‐old female patient presents with a chief complaint of cervical discomfort for the last 8 years, associated with progressive dysphagia when eating solids. She also experienced nighttime coughing, regurgitation, and intermittent hoarseness. Her past medical history was unremarkable, except for a left thyroid nodule resection for benign disease 6 years ago. The physical examination was normal. A contrast esophagogram was obtained and evidenced a 4‐cm left‐sided diverticulum protruding from the anterior‐lateral wall of the cervical esophagus (Figures 1 and 2). Upper endoscopy confirmed the previous finding and did not show other alterations.

**Figure 3:**
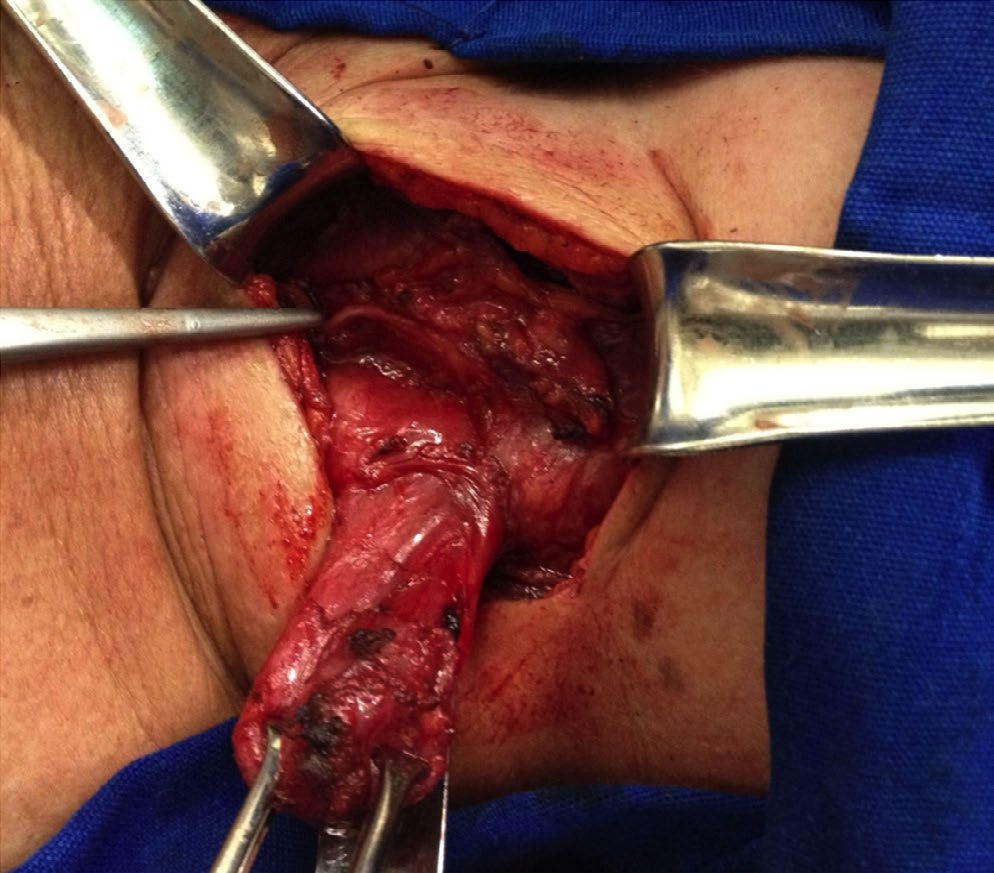
The diverticulum was dissected through a left cervicotomy. The recurrent laryngeal nerve was identified and preserved

**Figure 4:**
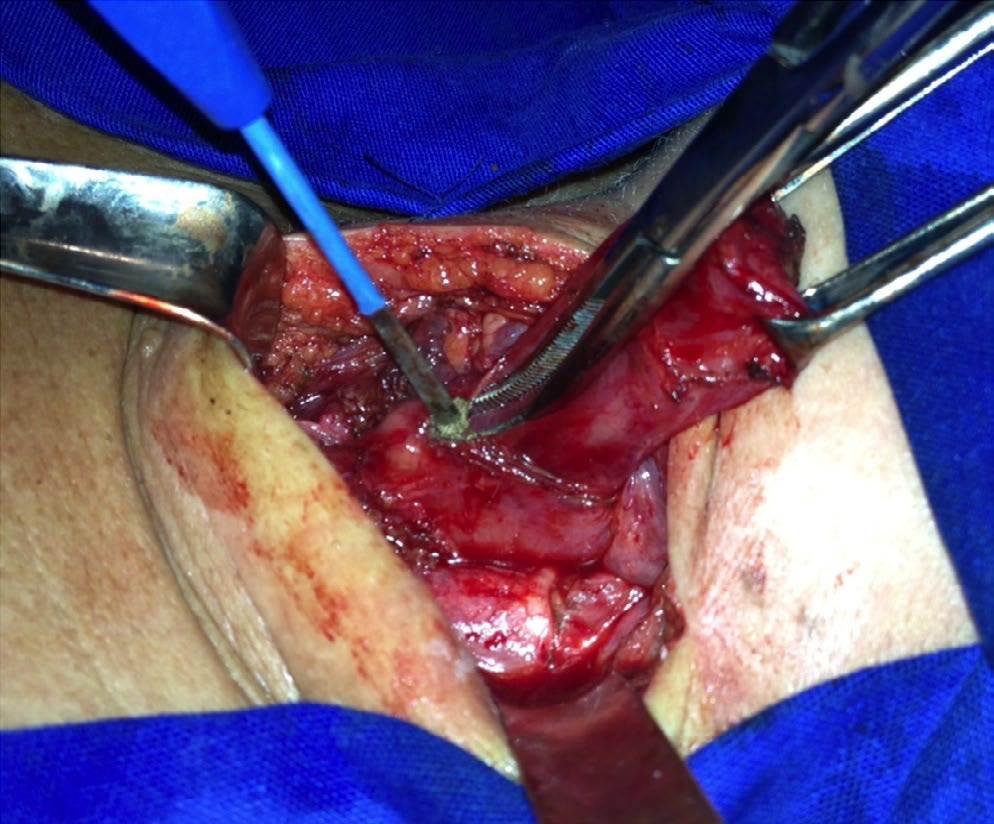
A 4‐cm miotomy of the cervical esophagus below de diverticulum was performed

**Figure 5:**
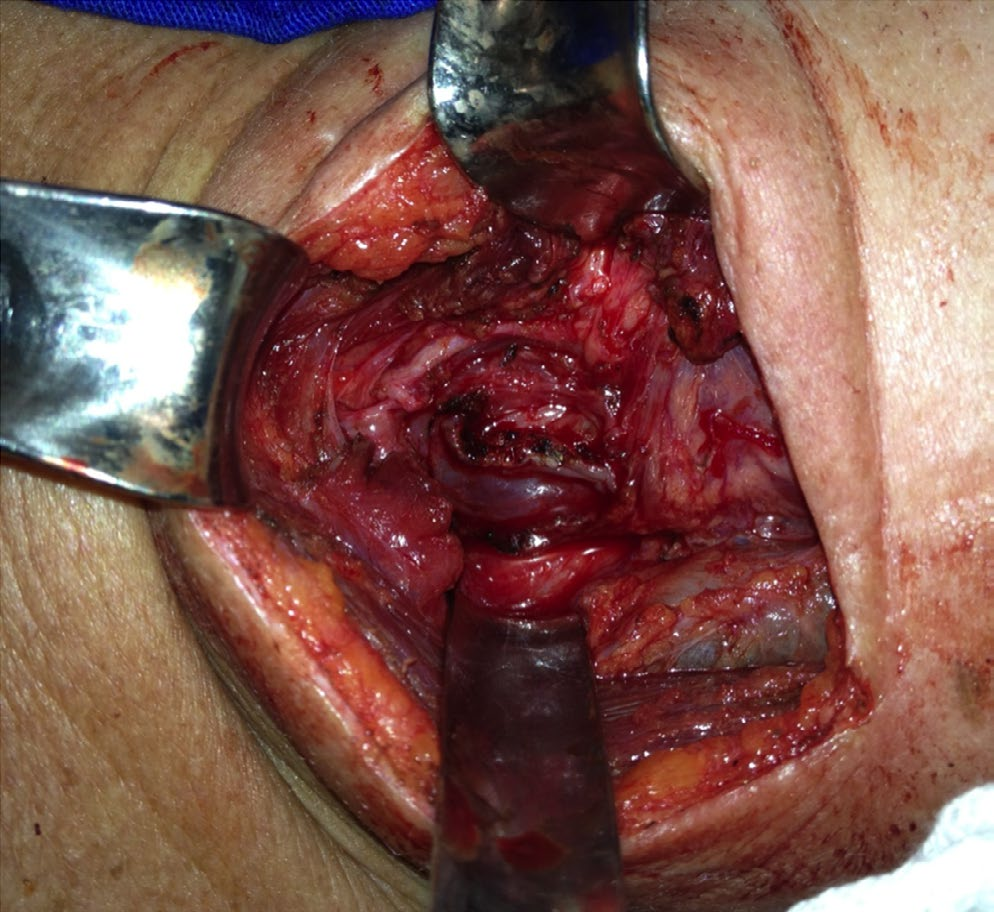
The diverticulum was resected by linear stapler

**Figure 6:**
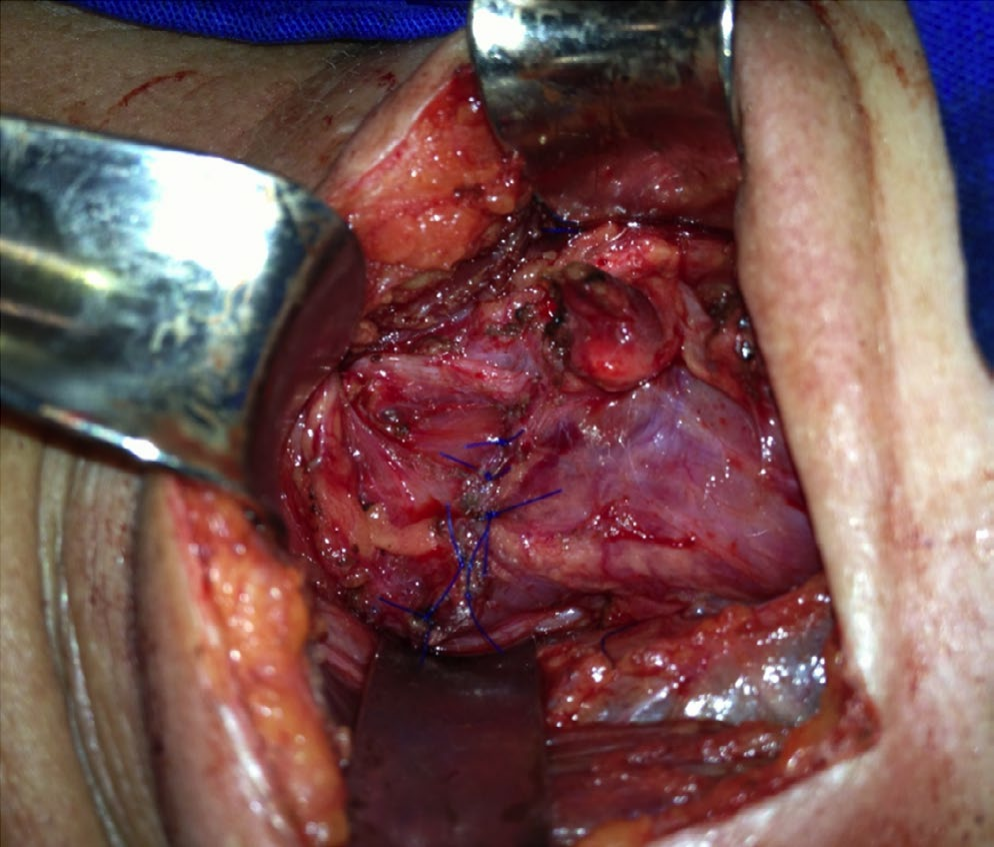
Final aspect after interrupted transverse sutures covering the stapling line

After consultation with the patient, a diverticulectomy and esophagomyotomy were scheduled. The diverticulum was approached through an oblique incision along the anterior border of the left sternocleidomastoid muscle. The diverticulum was dissected, with careful identification and preservation of the left recurrent laryngeal nerve, which was very close to its base (Figure 3). A 4‐cm esophagomyotomy was then performed (Figure 4). With a bougie in the esophagus, a linear stapler (Proximate TL‐30, Ethicon Inc) was used to transect the diverticulum base (Figure 5). The muscle gap was closed with interrupted transverse sutures covering the stapling line and avoiding esophageal stenosis (Figure 6). A nasogastric Dobhoff catheter was positioned, and the wound was then closed in layers with the placement of a Penrose drain. The postoperative course was uneventful. The patient was started on an enteral diet on postoperative day 1, and hospital discharge occurred on postoperative day 2. On postoperative day 7, the patient swallowed a methylene blue solution that did not reveal any leak. The drain and the nasogastric catheter were then removed, and an oral diet was initiated. The patient remains asymptomatic 3 months following surgery.

## 3 DISCUSSION

This study describes the surgical management of a patient with successfully treated KJD. KJD is a rare disease and has been described more frequently in the last years. Because of the unfamiliarity with this entity, we believe that KJD could be often misdiagnosed as a ZD by endoscopic or radiographic evaluation. Ekberg and Wahlgren^9^ analyzed 854 patients with dysphagia by pharyngoesophagographic examination; ZD was found in 20 patients (2.3%), and KJD was found in 16 patients (1.9%).

The pathogenesis of KJD is still unknown. Tang *et al* 7 hypothesized that KJD is the result of functional outflow obstruction in the proximal esophagus secondary to inappropriately constriction of its circular muscle fibers. This is similar to the most believed theory for the genesis of the ZD, which suggests a functional outflow obstruction of the pharynx due to cricopharyngeal dysfunction as its cause. The same authors also suggested that the symptomatic KJD could also result from esophageal dysmotility secondary to food residue–induced chronic inflammation within and around the diverticulum. Most of the KJDs are unilateral, but bilateral diverticula had been described in up to 25% of the cases.^10^, ^11^

Dysphagia is the most common symptom experienced by patients with KJD; however, asymptomatic patients have been described. Rubesin *et al* 10 described 16 patients with KJD; five of them were asymptomatic. Between the 11 symptomatic patients, eight had abnormal pharyngeal motility or an abnormal oral phase of swallowing, which may have contributed to or caused these symptoms. These motility abnormalities could also represent an etiopathogenic underlying phenomenon of the diverticula. Other presentation pictures of KJD include confounding findings with a thyroid nodule or mass on imaging studies^12^, ^13^, ^14^, ^15^ and, rarely, cervical celullitis.^16^

A barium pharyngoesophagogram usually establishes the diagnosis. A KJD is seen on the lateral wall of the pharyngoesophageal junction on anteroposterior view and below the cricopharyngeal muscle. That is different from the posterior outpouching above the cricopharyngeal muscle observed in patients with ZD.^17^ Eventually, a CT scan may be used to better locate the origin of the diverticulum, especially on patients’ large diverticula.^10^

There are few data regarding the treatment of KJD, and all the available literature consists of single‐case reports. Rogers *et al*,^18^ Kitazawa *et al*,^16^ and Undavia *et al* 19 performed surgical resection of the diverticulum without myotomy. Like our group, Boisvert *et al* 11 and Kim *et al* 20 were concerned with the underlying esophageal dysmotility and performed an esophagomyotomy associated with the diverticulectomy.

Successful endoscopic treatment was reported first by Tang *et al* 7 and then by Lee *et al*,^4^ with some technical modifications. The basic principle is not different from that for a ZD, namely, to dissect the septum between the diverticular sac and the esophagus. Because of the proximity of the inferior laryngeal nerves to the base of these diverticula, most groups will favor the transcutaneous route, although there were no cases of nerve injury described. Another important consideration that was not addressed until now is the risk of recurrence since the follow‐up of these patients is still short.

## 4 CONCLUSION

Killian‐Jamieson diverticulum is a rare entity that has been recognized more frequently at least in part because of its more extensive knowledge. Its etiopathogenesis and its treatment strategy are still unclear. Our group favors the cervical transcutaneous approach with diverticulum resection and associated esophagomyotomy for low‐risk patients. The endoscopic treatment should be an alternative, especially in high‐risk patients, despite the concern of inferior laryngeal nerve injury and long‐term results, which should be discussed with the patient. Studies regarding the etiopathogenesis and long‐term results of the treatment modalities employed until now must be encouraged.

## CONFLICT OF INTEREST

None declared.

## AUTHOR CONTRIBUTIONS

VBJ: analyzed and interpreted the data. ETB: acquired the data and drafted the article. SS and FCBdCS: drafted the paper. FT and AFD: revised the paper critically for relevant intellectual content. RAAS: conceived and designed the study. IC: made final approval of the version to be submitted.
